# Pneumocystis pneumonia in a child with sojia

**DOI:** 10.1186/1546-0096-12-S1-P225

**Published:** 2014-09-17

**Authors:** Marcel Schüller, Marie Macků, Jana Fráňová

**Affiliations:** 1 University Hospital Brno, Brno, Czech Republic

## Introduction

Pneumocystis pneumonia (PCP) is an opportunistic infection affecting patients with congenital or acquired immunodeficiency. In rheumatology, PCP is occasionally seen as a life threatening event complicating the course and treatment of a variety of diseases, especially vasculitides and systemic connective tissue disorders.

## Objectives

To show possible complications of the immunosuppressive treatment of systemic onset juvenile idiopathic arthritis (soJIA) and also demonstrate possible adverse effects and alternatives of antimicrobial treatment of PCP.

## Methods

A case report of a girl with soJIA and Pneumocystis infection.

## Results

A 6-year-old girl with soJIA treated with high doses of corticosteroids and cyclosporine for 6 months was sent to the Paediatric Rheumatology Centre at the Paediatric Department of the University Hospital Brno, Czech Republic. For lasting systemic symptoms (fever, rash, high inflammatory markers) and active knee arthritis the therapy with tocilizumab was initiated after discontinuation of cyclosporine. There was a history of repeated antibiotic and antifungal therapy for upper respiratory infections before starting the biological treatment. At the time of the first tocilizumab infusion, chest radiograph was negative, white blood cell count including neutrophils and lymphocytes was normal, the total IgG level was only slightly reduced. After the second dose of tocilizumab the corticosteroids could be tapered, but the girl presented with nonproductive cough and dyspnea. On the basis of typical symptoms, HRCT findings and highly positive PCR for Pneumocystis jiroveci in bronchoalveolar lavage the diagnosis of PCP was established and tocilizumab was discontinued. The first line PCP treatment with i.v. trimethoprim-sulfamethoxazole (TMP-SMX) for 17 days was successful, but it was complicated by agranulocytosis requiring application of colony stimulating factors and antibiotic treatment for febrile neutropenia. Granulocyte count was promptly normalized but simultaneously significant hypogammaglobulinemia occurred with the absence of CD20+ and significant decrease of CD19+ lymphocytes in peripheral blood, while the counts of T lymphocytes including CD4+ were normal. Monthly substitution by intravenous immunoglobulins (IVIGs) was required for six months until sustained normalization of plasma immunoglobulins was achieved. Intravenous pentamidine every 4 weeks has been used in the secondary PCP prophylaxis. The dose of corticosteroids could be significantly decreased because of inactivity of soJIA for a long period of expressed secondary immunodeficiency. However, eight months after setting the diagnosis of PCP the soJIA flared, possibly in connection with the restitution of the immune response. The therapy with anakinra was started with good response enabling corticosteroid withdrawal within 5 months. Now, the girl is without any difficulties, there are no clinical or laboratory signs of inflammation, her serum immunoglobulin levels are normal as well as the number of T-lymphocytes, even the B-lymphocyte count is almost normal. Secondary PCP prophylaxis still continues.

## Conclusion

Pneumocystis pneumonia in rheumatology should be considered particularly in patients with secondary immunodeficiency connected with aggressive and combined immunosuppressive therapy. However, no specific guidelines have been established for primary PCP prophylaxis in patients without HIV infection. PCP treatment with high doses of TMP-SMX may be associated with significant side effects such as agranulocytosis, which requires the use of second line drugs, for example pentamidine. Criteria for discontinuing the secondary PCP prophylaxis have also not been clearly defined and an individual approach should be used.

## Disclosure of interest

None declared.

